# Up-Regulation of Intestinal Vascular Endothelial Growth Factor by Afa/Dr Diffusely Adhering *Escherichia coli*

**DOI:** 10.1371/journal.pone.0001359

**Published:** 2007-12-26

**Authors:** Gaëlle Cane, Vanessa Liévin-Le Moal, Gilles Pagès, Alain L. Servin, Paul Hofman, Valérie Vouret-Craviari

**Affiliations:** 1 CNRS UMR 6543, Université de Nice-Sophia Antipolis, Nice, France; 2 Inserm, ERI-21, Faculté de Médecine de Nice, Nice, France; 3 Inserm, Unité 756; 4 Université Paris-Sud 11, Faculté de Pharmacie, Châtenay-Malabry, France; Ordway Research Institute, United States of America

## Abstract

**Background:**

Angiogenesis has been recently described as a novel component of inflammatory bowel disease pathogenesis. The level of vascular endothelial growth factor (VEGF) has been found increased in Crohn's disease and ulcerative colitis mucosa. To question whether a pro-inflammatory *Escherichia coli* could regulate the expression of VEGF in human intestinal epithelial cells, we examine the response of cultured human colonic T84 cells to infection by *E. coli* strain C1845 that belongs to the typical Afa/Dr diffusely adhering *E. coli* family (Afa/Dr DAEC).

**Methodology:**

VEGF mRNA expression was examined by Northern blotting and q-PCR. VEGF protein levels were assayed by ELISA and its bioactivity was analysed in endothelial cells. The bacterial factor involved in VEGF induction was identified using recombinant *E. coli* expressing Dr adhesin, purified Dr adhesin and lipopolysaccharide. The signaling pathway activated for the up-regulation of VEGF was identified using a blocking monoclonal anti-DAF antibody, Western blot analysis and specific pharmacological inhibitors.

**Principal Findings:**

C1845 bacteria induce the production of VEGF protein which is bioactive. VEGF is induced by adhering C1845 in both a time- and bacteria concentration-dependent manner. This phenomenon is not cell line dependent since we reproduced this observation in intestinal LS174, Caco2/TC7 and INT407 cells. Up-regulation of VEGF production requires: (1) the interaction of the bacterial F1845 adhesin with the brush border-associated decay accelerating factor (DAF, CD55) acting as a bacterial receptor, and (2) the activation of a Src protein kinase upstream of the activation of the Erk and Akt signaling pathways.

**Conclusions:**

Results demonstrate that a Afa/Dr DAEC strain induces an adhesin-dependent activation of DAF signaling that leads to the up-regulation of bioactive VEGF in cultured human intestinal cells. Thus, these results suggest a link between an entero-adherent, pro-inflammatory *E. coli* strain and angiogenesis which appeared recently as a novel component of IBD pathogenesis.

## Introduction

The two major forms of inflammatory bowel disease (IBD), Crohn's disease (CD) and ulcerative colitis (UC) have been defined on the basis of clinical, endoscopic and radiological criteria. The molecular pathogenesis of CD and UC appeared complex involving genetic susceptibility, modified innate or adaptative immune responses, multifactorial alterations in intestinal barrier function, mucosal homeostasis and the intestinal microflora [1]–[4]. Indeed, accumulative evidence suggests a role of resident intestinal microbiota or enterovirulent *E. coli* strains in initiation and pathogenesis of IBD. Particularly, studies highlighted a correlation between an increase in the number of biofilm-forming mucosal bacteria and pathogenesis of CD [5]–[7]. Identification of bacterial strains involved in etiology of IBD is not an easy task, however Tiveljung and co-workers have reported the presence of *E. coli*-, *Helicobacter spp*.-, *Mycobacterium paratuberculosis*- and *L. monocytogenes*-like 16S rDNA sequences in biopsies from the terminal ileum of CD patients [8]. *E. coli* have also been found abnormally predominant in early and chronic ileal lesions of CD patients [9]. Interestingly, healthy and ulcerated mucosa are colonized by *E. coli* strains having the same ribotype profile, which indicates uniform colonization regardless of the inflammatory state of the mucosa [10]. Finally, an adherent-invasive *E. coli* (AIEC) strain has been isolated from neoterminal ileum of CD patients [11]–[13]. Their virulence properties designate AIEC as a possible pathogen potentially able to induce persistent intestinal inflammation, by crossing and breaching the intestinal barrier, moving to deep tissues, promoting granulomas, continuously activating macrophages and producing inflammatory responses [14]–[18]. However, AIEC do not represent a specific pathogen exclusively found in CD because their presence was also observed in colonic control specimens.

Morphological, phenotypic and functional evidence of potent angiogenic activity in Crohn's disease (CD) and ulcerative colitis (UC) mucosa has been recently reported by Danese and co-workers [19]. In particular, an increase in microvessel density, associated to the presence of bioactive vascular endothelial growth factor (VEGF), was observed in inflammatory bowel disease (IBD). VEGF is not only the most potent angiogenic factor but has also been described as a mediator of tumor-associated immunodeficiency through inhibition of T-cell development [20]. Angiogenesis involves the recruitment and proliferation of endothelial cells from pre-existing vessels or circulating endothelial progenitor cells originating from the bone marrow [21]. The pleiotropic factor VEGF is secreted by tumor and stromal cells and regulates endothelial cell survival, proliferation, migration but also, allows the assembly of endothelial cells into capillary like structures (for reviews see [22], [23]).

In this study, we sought to determine if and how an entero-adherent pro-inflammatory *E. coli* could regulate the expression of VEGF in human intestinal epithelial cells. To do this, we focused our attention on Afa/Dr diffusely adhering *E. coli* (Afa/Dr DAEC) strains which belong to class six of the enterovirulent *E. coli* strains [24]. These strains of *E. coli* express a family of adhesins that are encoded by a family of operons consisting of at least five genes including the *afa*, *dra* and *daa* genes (for review see [25]). A recent epidemiological study demonstrates that bacteria expressing Afa adhesins adhere to the mucosa of patients with IBD [26]. Through Afa/Dr adhesins, these bacteria interact with membrane-bound receptors including the decay-accelerating factor (DAF/CD55) (Afa/Dr DAEC_DAF_) and members of the human carcinoembryonic-antigen-related cell-adhesion molecules (CEACAMs) family (Afa/Dr DAEC_CEACAMs_). It was previously demonstrated that the Afa/Dr DAEC strain C1845 [27] is an entero-adherent, pro-inflammatory *E. coli* that activates the extracellular regulated kinase/mitogen-activated protein kinases (Erk/MAP kinase) signaling pathway in human intestinal T84 cell monolayers promoting interleukin 8 (IL8) secretion and the transepithelial migration of polymorphonuclear neutrophils (PMNs), a feature of chronic inflammation [28]. In turn, the activated PMNs promote the production of tumor necrosis factor-α (TNFα) and interleukin-1β (IL1-β) that lead to the over-expression of DAF at the apical and basolateral domains of T84 cells [29]. Interestingly enough, DAF is upregulated in UC [30]–[32]. Moreover, in cultured Caco-2 cells specific interaction between bacterial adhesin AfaE-III and DAF leads to increased expression of MHC class 1 related molecule (MICA) suggesting that this host-bacteria interaction pathway could play a role in the pathogenesis of IBD since increased-MICA expression has been found at the surface of colonic epithelial cells in CD-affected patients [33]. Very recently, *E. coli* strain of the B2+D phylogenetic groups of *Enterobacteriaceae* have been found more likely to be associated with tissues from patients with UC and CD than from controls [34]. This phylogenetic group express a functional serine protease autotransporter (SPATE) and adherence factors. Interestingly, Afa/Dr DAEC express SPATE, also named Sat, and Sat is involved in the Afa/Dr DAEC-induced breach of the intestinal epithelial barrier [35].

In this study we show that Afa/Dr DAEC C1845 bacteria, following the recognition of DAF by the F1845 adhesin, activate Akt- and the extracellular signal-regulated kinase (Erk)-signaling pathways through a Src protein kinase-dependent mechanism, and induce a gene transcription program leading to the up-regulation of VEGF expression.

## Results

### Wild-type C1845 bacteria increase the production of VEGF in cultured human intestinal T84 cells

We performed experiments on the human colonic carcinoma T84 cell line that maintains the morphological and functional characteristics of epithelial tissues [36] and that has been widely used in the fields of inflammation and infection [37]. Bacterial infection, with a multiplicity of infection of 20 bacteria per epithelial cells, was performed at 37°C on polarized, starved T84 epithelial cells. Careful microscopic observation of cells after infection with control or wild-type C1845 bacteria did not reveal any cell toxicity (data not shown). We tested by q-PCR the expression of genes encoding for VEGF and for the pro-inflammatory cytokine IL8, which is used here as a control of the wild type C1845–induced pro-inflammatory responses. Results show in table 1 that VEGF and IL8 genes are up-regulated by 3.6 and 1.7-fold, respectively, in wild-type C1845-infected T84 cells as compared with non-infected cells. Observation that the genes coding for Transforming growth factor Receptor 2 (TGFβ R2), Epithelial growth factor receptor 1 (EGF R1) and placental growth factor (plGF) are not modulated indicate that the up-regulation of VEGF and IL8 genes do not result of a general up-regulation program in wild-type C1845-infected T84 cells. This result highlights the capacity of wild-type C1845 bacteria to specifically regulate the expression of genes coding for a major angiogenic factor and a pro-inflammatory cytokine.

**Table 1:** Wild-type C1845-induced gene expression

Genes (assay ID)	Fold induction
VEGF (Hs00173626_m1)	3.60±0.45 (n = 4)*
IL8 (Hs00174103_m1)	1.70±0.16 (n = 3)*
PlGF (Hs00182176_m1)	1.00±0.16 (n = 3)*
TGFβ R2 (Hs00234253_m1)	0.78±0.21 (n = 2)
EGF R1 (Hs00193306_m1)	0.70 (n = 2)

Cells were infected for four hours with Afa/Dr DAEC wild-type strain C1845 and total RNA extracted. Selected gene expression was assayed by q-PCR. The oligonucleotides used in this study were designed by Applied Biosystems, the manufacturer's references are given in brackets. Abbreviations are as follows: VEGF: Vascular Endothelial Growth Factor, IL8: interleukin 8, PlGF: Placental growth factor, TGFβ R2: Transforming growth factor Receptor 2, EGF R1: Epithelial growth factor Receptor 1. Results (means±SD) are representative of independent experiments (where n represents the number of experiments). *, p<0.001 versus non infected cells.

**Figure 1:**
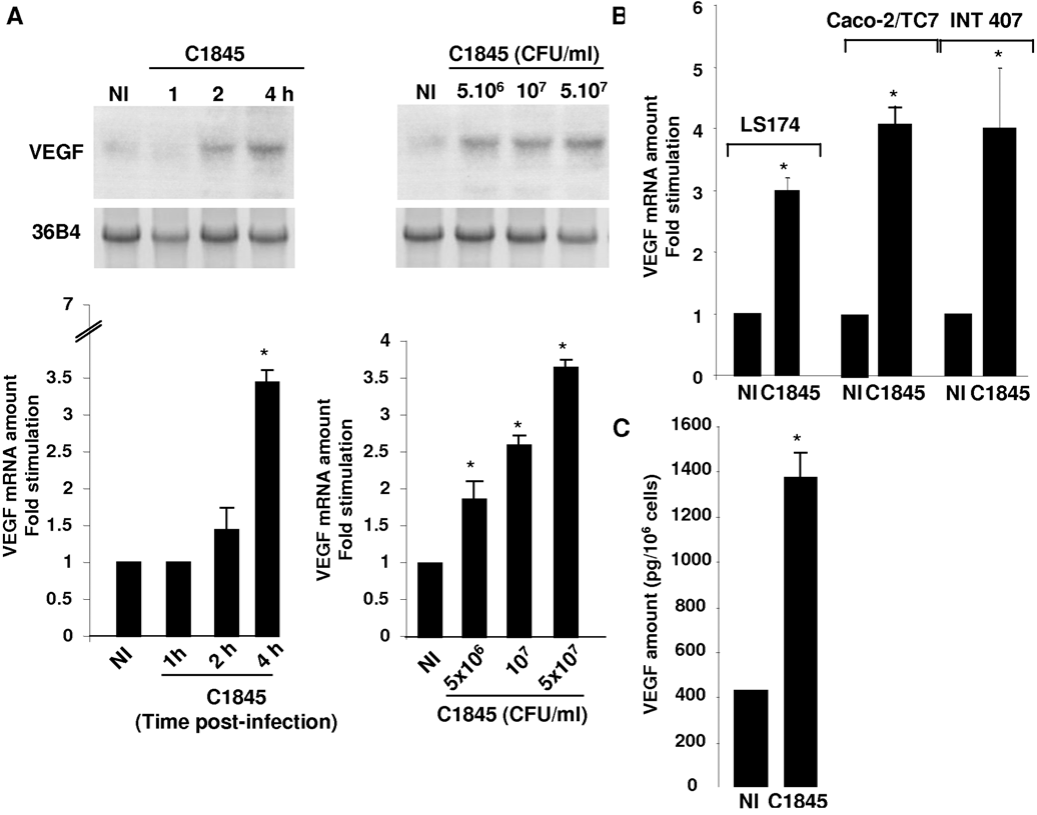
C1845 bacteria increase VEGF expression in cultured intestinal epithelial cells. In A, increase in VEGF mRNA expression. Confluent serum-starved T84 cells (5×10^6^ cells/well) were infected with wild-type C1845 bacteria and total RNA was prepared as indicated in the Materials and Methods section. Results for non-infected (NI) or cells infected with 5×10^7^ CFU/ml C1845 bacteria for the indicated time are shown on the left panel. The dose response effect is presented on the right. Cells were infected for four hours with the indicated number of bacteria. Results of Northern blots are presented in the upper part of the Figure and q-PCR shown in the lower part. These results are representative of three independent experiments. In B, confluent serum-starved LS174, Caco-2/TC7 or INT407 cells were infected with 5×10^7^ CFU/ml wild-type C1845 bacteria for four hours. VEGF mRNA expression was assayed by q-PCR. The signal corresponding to VEGF and 36B4 transcripts was quantified using a phosphoImager. Under each condition the signal was normalized to the 36B4 probe. Results are expressed as arbitrary units corresponding to the fold stimulation of treated versus non-treated conditions. In C, increase in the VEGF protein in the culture medium of wild-type C1845-infected T84 cells. Cells were infected with 5×10^7^ CFU/ml wild-type C1845 bacteria for four hours and the supernatant were collected as indicated in the Materials and Methods section. The VEGF protein level was then quantified using an ELISA. Quantification of the results from two independent experiments (means±SD) is shown. *, p<0,01

Northern blot analysis of total RNA using a specific probe for the VEGF gene shows a very low level of VEGF mRNA expression in control, non-infected T84 cells while a time and dose-dependent increase in VEGF mRNA expression was observed in T84 infected cells (Figure 1A, upper panel). The transcript of 4 kb is preferentially expressed, however the expression of the three other spliced variants, ranging from 1.5 to 4.0 kb, is revealed with a longer exposure of the autoradiogram (data not shown). VEGF mRNA expression in non-infected and wild-type C1845-infected T84 cells was also quantified by q-PCR (Figure 1A, lower panel). The maximum increase in VEGF mRNA expression (3.6-fold increase) was achieved when cells were infected with 5×10^7^ CFU/ml of wild-type C1845 bacteria for four hours. This increase in VEGF mRNA expression in response to wild-type C1845 bacteria is neither cell line-dependent, since we observed the same effect in the human LS174 colon cell line and the fully-differentiated enterocyte-like Caco-2/TC7 cells nor transformation-dependent since bacteria increase the expression of VEGF in INT407, a non-transformed cell line (Figure 1B).

In order to examine the effect of C1845 bacteria on VEGF production, we assayed the levels of secreted VEGF by an ELISA, in culture medium of non-infected and wild-type C1845-infected T84 cells. As shown in Figure 1C, C1845-infected cell supernatant contains 3.4-fold more VEGF protein than the control supernatant of non-infected cells.

**Figure 2:**
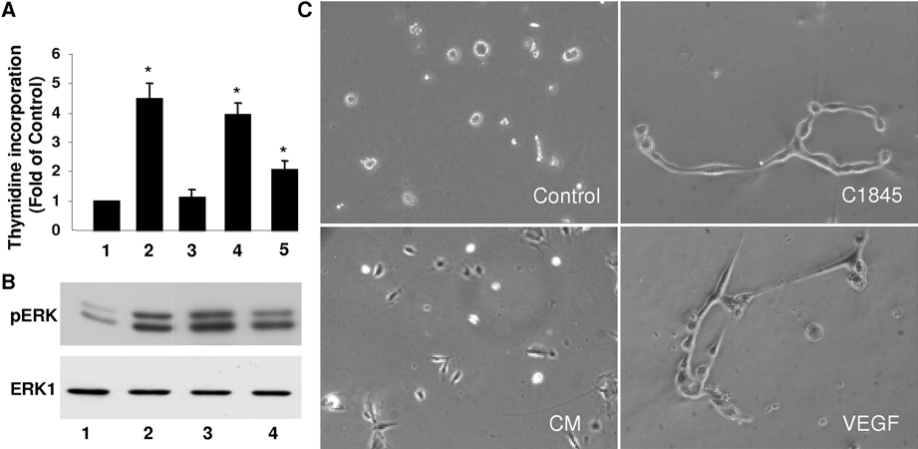
Proliferative response, Erk activation and tube like structure formation in HUVEC in the presence of culture medium of wild-type C1845-infected T84 cells. ^3^H-thymidine incorporation into DNA and Erk activation were essayed in HUVEC incubated with the following. In A, ^3^H-thymidine incorporation into DNA. Control (1), VEGF at 10 ng/ml (2), culture medium of non-infected T84 cells (3), culture medium of wild-type C1845-infected T84 cells (1×) (4), culture medium of wild-type C1845-infected T84 cells (0.5×) (5). In B, Western blot showing phospho-Erk1/2 and total Erk1/2 visualized with respectively anti-phospho-Erk and anti-Erk antibodies. Control (1), VEGF at 10 ng/ml (23), culture medium of wild-type C1845-infected T84 cells (1×) (3), culture medium of wild-type C1845-infected T84 cells (0.5×) (4). The results are representative of at least three independent experiments. *, p<0,01. In C, Endothelial cells were seeded on matrigel and incubated with growing medium (control) alone or supplemented with T84 conditionned medium (CM), C1845–condition medium (C1845) or VEGF. Images show that only C1845-conditionned medium (C1845) and VEGF allow the alignment of endothelial cells in tube like structure.

To further demonstrate that the VEGF present in culture medium of wild-type C1845-infected T84 cells, was biologically functional, we used three different tests in Human Umbilical Vein Endothelial Cell (HUVEC): the determination of ^3^H-methyl thymidine incorporation into DNA, an index of cell proliferation, activation of extracellular regulated kinase (Erk) and the formation of tube like structures in matrigel [38]. As shown in Figure 2A, VEGF contained in the culture medium of wild type C1845-infected T84 cells (bars 4 and 5) increases thymidine incorporation into HUVEC DNA to the same extend than recombinant human VEGF (bar 2). By the same way, conditioned medium of wild type C1845-infected T84 cells triggers the phosphorylation of Erk1/2 in HUVEC (Figure 2B, lanes 3 and 4) as does recombinant VEGF (lane 2). And finally, only the medium conditioned by C1845 bacteria is able to induce the formation of tube like structures in matrigel (Figure 2C). We conclude from these experiments that VEGF produced by intestinal cells challenged with wild type C1845 bacteria is fully active.

### VEGF production induced by wild-type C1845 bacteria depends on DAF activation

**Figure 3:**
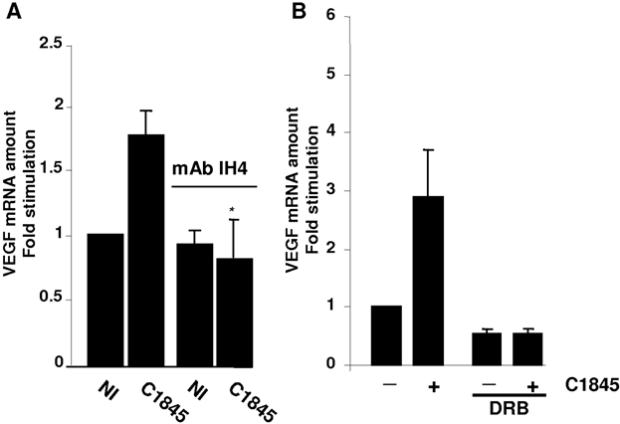
Increase in VEGF mRNA by wild-type C1845 bacteria requires binding to brush border-associated DAF. In A, binding to DAF is necessary for the C1845-induced increase in VEGF mRNA expression. Confluent serum-starved T84 cells infected were with wild-type C1845 at 5×10^7^ CFU/ml, which represents a multiplicity of infection of 20 bacteria per epithelial cell, for two hours. When indicated, cells were treated for 30 minutes prior to infection with the anti-DAF blocking IH4 monoclonal antibody. VEGF mRNA expression was assayed by q-PCR. *, p = 0,03 (n = 7). In B, wild-type C1845-induced VEGF mRNA increase is blocked by transcription inhibitor. Confluent serum-starved T84 cells were pre-treated for 30 minutes with 25 µg/ml of the reversible transcription inhibitor, 5,6-dichloro-1-beta-D-ribobenzimidazole (DRB). Cells were then infected with 5×10^7^ CFU/ml wild-type C1845 for four hours and total RNA prepared. The relative quantity of VEGF mRNA was measured by q-PCR. Quantification of the results from three independent experiments (means±SD) is shown. *, p<0,01.

We examined whether the interaction of wild-type C1845 bacteria with its membrane-bound receptor, the DAF [39], triggers the up-regulation of VEGF mRNA expression. T84 cells were pre-treated for 1 hour with the IH4 blocking monoclonal antibody and then infected for 2 hours with wild-type C1845 bacteria. As shown in Figure 3A, the levels of VEGF mRNA in non-infected or non-infected and IH4-treated cells are not significantly different, indicating that the antibody by itself does not interfere with VEGF mRNA expression. In the wild-type C1845-infected cells, the 1.8-fold increase in the level of the VEGF mRNA is fully blocked when cells are pre-treated with the IH4 antibody. This result clearly demonstrates that activation *via* DAF is a pre-requisite for signaling leading to VEGF production.

An increase in the VEGF mRNA level could be due to a transcriptional and/or a post-transcriptional effect. We used the transcriptional inhibitor 5, 6-dichloro-1-beta-d-ribofuranosylbenzimidazole (DRB), which reversibly inhibits RNA polymerase II to test whether the increase in VEGF mRNA is linked to a transcriptional mechanism. As shown in Figure 3B, when the non-infected T84 cells are pre-treated with DRB, the basal level of VEGF mRNA decreased by two-fold. This indicates that the VEGF gene is transcribed at a basal level. Treatment of the wild type C1845-infected cells with DRB totally abolished the increase in VEGF mRNA expression. Yet, we conclude that the induction of VEGF mRNA by wild type C1845 bacteria is largely dependant on transcriptional regulation.

### A member of the Src kinase family, Akt and Erk are involved in wild type C1845-induced VEGF production

**Figure 4:**
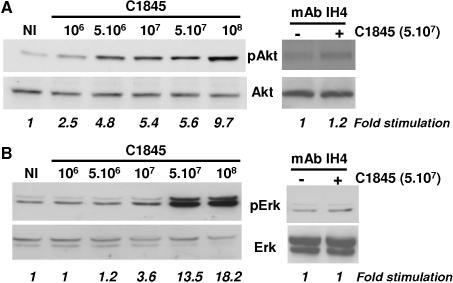
Wild-type C1845 bacteria induce Akt and Erk signaling pathways in T84 epithelial cells. Confluent serum-starved T84 cells were non-infected (NI) or infected with increasing concentrations of wild-type C1845 bacteria, for two hours. When indicated, cells were pre-treated with an anti-DAF blocking IH4 monoclonal antibody prior to or in the absence of infection. In A, phospho-Akt and total Akt were assayed by Western blotting using respectively anti-phospho-Akt and anti-Akt antibodies. In B, phospho-Erk1/2 and total Erk1/2 were visualized with respectively anti-phospho-Erk and anti-Erk antibodies. These results are representative of at least three independent experiments.

Having demonstrated that DAF recognition is necessary to transmit signals from the membrane to the nucleus, we further characterized which signaling pathways are involved in wild type C1845-induced VEGF production. Given that in most cell lines the Akt and Erk pathways are largely involved in VEGF production [40], we tested whether wild-type C1845 bacteria activate these kinases. T84 cells were infected with increasing doses of wild-type C1845 bacteria for 2 hours and cell proteins were extracted. The Akt or Erk activities were assayed by Western blotting with antibodies against the active phospho-proteins. Both Akt (Figure 4A) and Erk (Figure 4B) were activated following wild-type C1845 infection. The maximum of both Akt and Erk activation was observed at a concentration of 10^8^ CFU/ml of wild-type C1845 bacteria, which represents a multiplicity of infection of 50 bacteria per epithelial cell, with respectively 9.7 and 18.2-fold stimulation. Interestingly, the activation of both kinases is inhibited when the wild-type C1845-infected cells were pre-treated with the DAF blocking monoclonal antibody IH4.

**Figure 5:**
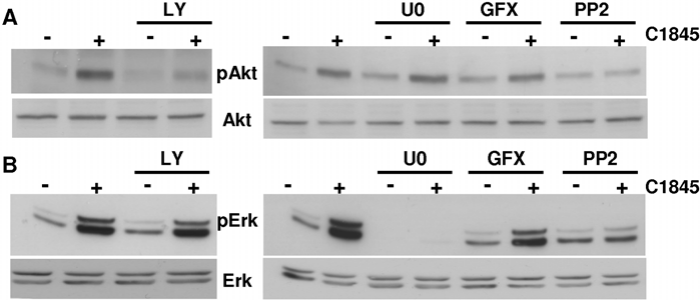
A Src protein is involved in wild-type C1845-induced Akt and Erk activation. Confluent serum-starved T84 cells were pre-treated with 15 µM LY294002 (LY), 10 µM U0126 (U0), 5 µM GF109203X (GFX) or 5 µM PP2 for 30 minutes prior to infection or no infection with wild-type C1845 bacteria at 5×10^7^ CFU/ml for two hours. Cells were lyzed in SDS sample buffer and phospho-Akt and total Akt (in A) and phospho-Erk and total Erk1/2 (in B) assayed by Western blotting as described in Figure 5. These results are representative of at least three independent experiments.

In INT 407 epithelial cells, it was observed previously that wild-type C1845 bacteria promote a protein kinase C (PKC) activation [41]. We used GF 109203X (GFX), a selective cell-permeable inhibitor of the PKCα, β_I_, β_II_, γ, δ and ε isoforms, to determine whether PKC could regulate the wild-type C1845-induced Erk and/or Akt pathways. Pre-treatment of T84 cells with GFX did not significantly modify either the basal level or the C1845-stimulated level of Akt (Figure 5A) or Erk (Figure 5B). Thus, in response to DAF recognition PKC is not involved in the regulation of Akt and Erk in C1845-infected T84 epithelial cells. As expected, we found that Akt- and Erk-activation were fully blocked by the PI3K inhibitor (LY294002) and the MEK inhibitor (U0126), respectively (Figure 5A and B).

We sought to determine the molecular intermediates acting between the DAF receptor and these two signaling pathways. It has been shown that the GPI-anchored DAF has signal transduction capabilities. Since cross-linked DAF has been shown to associate with p56lck and p59fyn in cells [42], we tested the hypothesis that a Src kinase could be implicated in wild-type C1845-induced signaling pathways. T84 cells were pre-treated with the PP2 inhibitor of Src kinase and the Akt or Erk activity was assayed by Western blotting. As shown in Figure 5A and B, in the presence of PP2 both the wild-type C1845-induced increase of Akt and Erk activities were inhibited in infected cells as compared with the non-infected cells. This demonstrates that a Src protein kinase is activated downstream of DAF recognition by wild-type C1845 bacteria and is necessary to transduce the signal to Akt and Erk.

**Figure 6:**
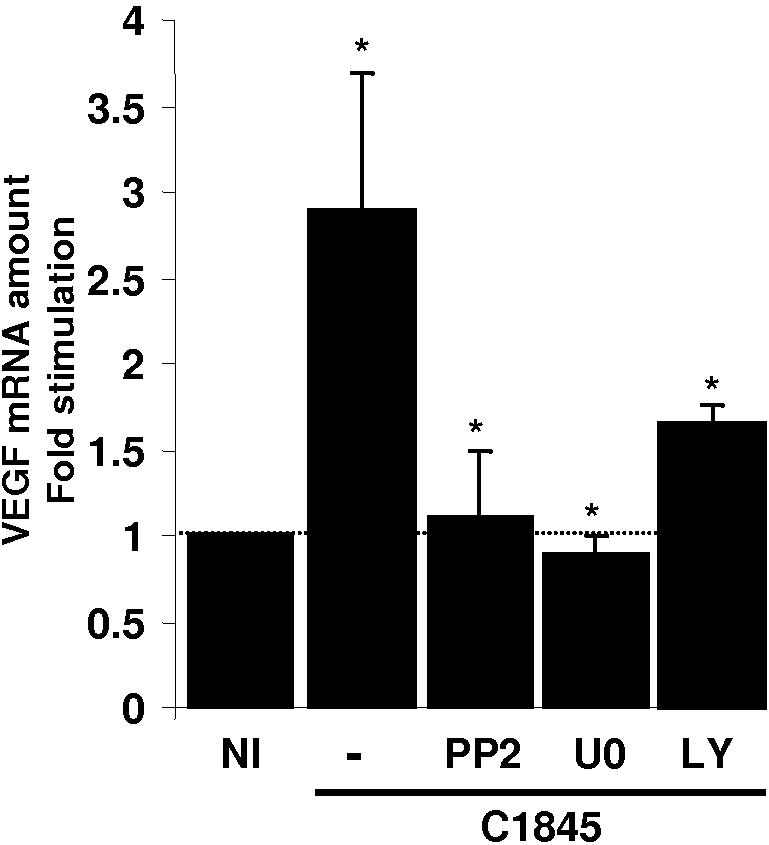
Wild-type C1845-induced VEGF expression is dependant on Src and Erk proteins. Confluent serum-starved T84 cells were pre-treated with 5 µM PP2, 10 µM U0126 or 15 µM LY294002 for 30 minutes. Cells were then infected with wild-type C1845 bacteria at 5×10^7^ CFU/ml for four hours. Total RNA was prepared and the relative quantity of VEGF mRNA was measured by q-PCR. Quantification of the results from four independent experiments (means±SD.) is shown. *, p<0.005 versus non infected cells.

Using this pharmacological approach, we then verified whether these pathways are required for VEGF mRNA expression in response to wild type C1845 bacteria. Prior to C1845 infection, T84 cells were pre-treated with the different inhibitors used above. Total RNA was extracted and the level of the VEGF transcripts was assayed by q-PCR. As shown in Figure 6, when the C1845-infected cells are pre-treated with the Src inhibitor, PP2, the increase in VEGF mRNA was inhibited. Interestingly, we observed the same result when the C1845-infected cells were treated with the MEK inhibitor, U0126 whereas the PI3K inhibitor partially blocked the production of the VEGF transcript in wild type C1845-infected cells. This partial inhibition suggests that VEGF transcription is regulated by at least two distinct signalling pathways in which Erk activation is a pre requisite for PI3K/Akt effect on VEGF promoter. We conclude that a Src-dependant Erk pathways is required to increase VEGF mRNA expression in response to C1845 bacteria.

### F1845 adhesin is the bacterial factor involved in VEGF up-regulation

**Figure 7:**
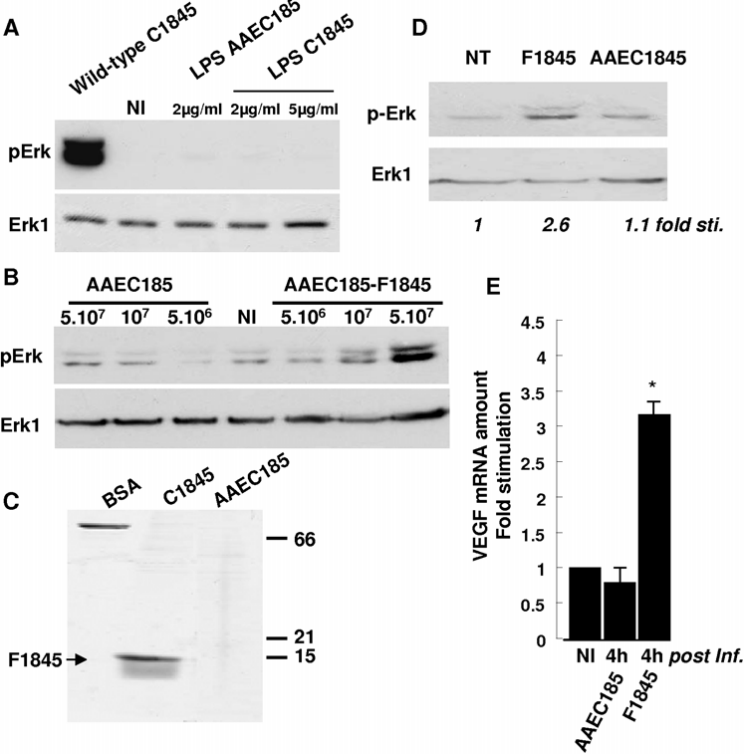
F1845 adhesin but not LPS, activates Erk and increases VEGF expression. In A, confluent serum-starved T84 cells were non infected (NI), infected with 5×10^7^ CFU/ml wild-type C1845 bacteria, or treated for 2 hours with indicated concentration of LPS purified from non-pathogenic AAEC185 or C1845 strains. Phospho-Erk1/2 and total Erk1 were visualized by Western Blotting with respectively anti-phospho-Erk and anti-Erk1 antibodies. This experiment is representative of two independent experiments. In B, confluent serum-starved T84 cells were non-infected (NI), infected with increasing concentrations of AAEC185 or recombinant AAEC185-F1845 strains for 2 h. Phospho-Erk and total Erk-1 were assayed by Western blotting using respectively anti-phospho-Erk and anti-Erk1 antibodies. In C, coomassie-stained polyacrylamide-SDS gel of fimbriae purified from C1845 or AAEC185 bacteria. 1 µg of bovine serum albumine (BSA) are showed on the left of the gel. In D, confluent serum-starved T84 cells were non treated (NT) or incubated 2 h in the presence of extracts of C1845 bacteria (containing 2 µg of purified F1845 adhesin) or AAEC185 bacteria. These result are representative of three independent experiments. In E, confluent serum-starved T84 cells (5×10^6^ cells/well) were infected with AAEC185 bacteria or recombinant AAEC185-F1845 bacteria expressing the pSSS1 operon encoding the F1845 adhesin (5×10^7^ CFU/ml). Total RNA were extracted and analysed by q-PCR. These results are representative of at least two independent experiments (means±SD).

We further conduct experiments in order to identify the bacterial factor that was responsible for the up-regulation of VEGF in intestinal cells. DAF has been demonstrated to be part of the lipopolysaccharide (LPS)-induced receptor complex [43]. Despite the fact that T84 cells have been described previously to be completely unresponsive to LPS [44], we wanted to clarify this point. We purified LPS from the non-pathogenic strain AAEC185 and from the wild-type strain C1845 and examined whether these purified LPS could induce signaling pathways on T84 cells. As shown in Figure 7A, up to 5 µg/ml of LPS purified from wild-type C1845 or non-pathogenic AAEC185 bacteria were not able to induce an increase in Erk activity, or VEGF production (data not shown), whereas wild-type C1845 bacteria efficiently increase the level of phosphorylated forms of Erk (Figure 7A).

We further checked that F1845 adhesin is involved in the induction of Erk signaling pathway. T84 cells were infected with non-pathogenic strain AAEC185 or recombinant *E. coli* AAEC185-F1845 strain expressing the pSSS1 plasmid encoding the F1845 adhesin, and Erk activity was assayed by Western blotting using an anti phospho-Erk antibody. As shown in Figure 7B, only the recombinant AAEC185-F1845 strain, increases the phosphorylation of Erk. We also performed the same experiment with the F1845 adhesin purified from wild-type strain C1845 (Figure 7C). As shown in Figure 7D, two micrograms of the purified F1845 adhesin were able to stimulate Erk activity inT84 cells.

We further controlled whether the F1845 adhesin increases the VEGF mRNA expression in T84 cells. For this purpose, we used the non-pathogenic *E. coli* AAEC185 and the recombinant AAEC1845-F1845 strain. When the cells were infected for four hours with the recombinant AAEC1845-F1845 strain, we observed a 3.2-fold increase in VEGF mRNA expression (Figure 7E). In contrast, when the cells were infected for the same time with the control AAEC185 strain no increase in the level of VEGF mRNA expression was observed. These results indicate that the F1845 adhesin expressed by recombinant strain *E. coli* AAEC185-F1845 induces the up-regulation of VEGF expression in T84 human intestinal epithelial cells.

## Discussion

For many years it was suspected that increased VEGF expression and angiogenesis were associated with IBD [45]–[50]. In particular, during IBD the density of the microvasculature of the colon is increased allowing an increase in leukocyte recruitment and subsequent tissue damage. This observation leads to the concept that angiogenesis may play a pathological role in IBD [51]. The ultimate demonstration that a link between IBD and angiogenesis exits arise from two recent observations: vascular remodeling and functionally active angiogenesis are associated with IBD [52] and the angiogenic blockade, using the anti-angiogenic peptide ATN-61, has decreased angiogenesis in two colitis-associated mice models [19].

Interestingly, although serum VEGF levels and angiogenesis are increased in IBD and correlate with disease activity, they do not appear genetically determined [53], suggesting that external factors are involved in VEGF up-regulation. Examining the cellular sources of VEGF in IBD mucosa, it was found that lamina propria mononuclear cells stimulated or not by LPS and human intestinal fibroblasts stimulated with TNF-α produce VEGF, whereas unstimulated or TNF-α-stimulated intestinal microvascular endothelial cells produce negligible amount of VEGF [19]. Here, we demonstrate that the human intestinal epithelial cells are a novel source of VEGF when challenged by the entero-adherent, pro-inflammatory Afa/Dr DAEC C1845 strain.

We identified in T84 human intestinal cells the mechanism by which VEGF is up-regulated in response to wild type C1845 strain. The process of induction implies the recognition of a membrane-bound receptor DAF by the bacterial F1845 adhesin. DAF, the receptor for Afa/Dr_DAF_ adhesions [39], [54], is associated *via* its GPI-anchor with lipid rafts, sphingolipids and signaling proteins [55]. In intestinal and non-intestinal epithelial cells, DAF is clustered around adhering Afa/Dr bacteria [56], [57]. As for the majority of GPI-anchored proteins, the signaling intermediates that link DAF to signaling pathways are not yet fully characterized. However, in T-cells, cross-linked-DAF becomes tyrosine phosphorylated and is associated with the p56^lck^ and p59^fyn^ protein tyrosine kinases. Here, using a blocking mAb directed against DAF and the PP2 compound, a large spectrum inhibitor of the Src family protein tyrosine kinases, we demonstrate that the up-regulation of VEGF was a DAF- and Src kinase-dependent mechanism. Downstream the activated Src family kinase(s), we identified the Erk and Akt signaling pathways as involved in the C1845-induced up-regulation of VEGF. Angiogenesis a consequence of increasing expression of VEGF through the activation of Erk and PI3K/Akt pathways has been previously described in relation with progression of tumors but not in IBD. For examples, PI3K/Akt and Erk signaling pathways are involved in bcl-2-induced VEGF expression in melanoma cells [58]. VEGF up-regulation via both PI3K and Erk pathways occurs in fibroblasts [59], [60]. Up-regulation of VEGF by Gram negative [61]–[67] and Gram positive [68]–[70] bacteria has been previously reported. Gastrointestinal pathogens including *Helicobacter pylori* [71]–[73] and *Listeria monocytogenes* [74] increased the expression of VEGF mRNAs through a Erk-mediated pathway, as we found here for the wild-type Afa/Dr DAEC strain C1845. It was noticed that *H. pylori* [75], as Afa/Dr DAEC [39], [54], use DAF as a receptor.

In the light of the recent findings that angiogenesis might contribute to the initiation and perpetuation of IBD, our results showing that an entero-adherent, pro-inflammatory bacteria has the capacity to induce the up-regulation of the most potent angiogenic factor VEGF, are of major interest. It was recently proposed that Afa/Dr DAEC is a “silent pathogen” that can emerge from the human intestinal microbiota (for review see [25]). Whether the adhesin-dependent signaling pro-inflammatory events induced by Afa/Dr DAEC in different cultured human intestinal cell lines observed here and previously [28], [29], [33] are linked in some way, to chronic inflammation in vivo remains to be determined. Formal demonstration requires further *in vivo* experiments in appropriate humanized animal models since the receptors for Afa/Dr DAEC, DAF [76] and human CEACAMs (A. Servin, personal communication), are highly specific of human. In addition, it remains to be determined whether other entero-adherent *E. coli* strains and AIEC strains isolated from IBD mucosa are able to up-regulate VEGF expression in intestinal cells.

## Materials and Methods

### Reagents and antibodies

All reagents, unless specified, were from Sigma Chemicals (Saint Quentin Fallavier, France). Tissue culture plastic ware was from Nunc (Roskilde, Denmark). The Src family kinase inhibitor PP2 [4-amino-5-(4-chlorophenyl)-7(t-butyl)pyrazololol[3,4-d]-pyrimidine] and the Protein Kinase C inhibitor GF109203X (bisindolylmaleimide 1) were from Calbiochem. The MAP/Erk Kinase (MEK) inhibitor U0126 was from Promega (Madison, WI, USA). Anti Akt and anti-phospho Akt (pS473) polyclonal antibodies were from Cell Signaling Technology (Beverly, MA). The rabbit polyclonal anti-Erk antibody (E1B4) that preferentially recognizes Erk2 was raised against a C-terminal Erk1 peptide. The anti-phospho Erk antibody (clone MAPK-YT) and anti alpha-tubulin antibody were from Sigma. The IH4 antibody was a gift from D.M. Lublin (Washington University, St. Louis). This mAb is specific of CCP3 domain of DAF [77]. It has previously been used to map the Dr adhesin binding site to hDAF and its block the complement regulatory function of DAF [57], [78].

### Bacterial strains

We used the wild type Afa/Dr DAEC C1845 [27], harboring fimbrial F1845 adhesin, and as negative control the non-pathogenic AAEC185 *E. coli* strain [79]. The bacteria were grown overnight at 37°C on LB-agar plates and expanded in LB medium at 30°C without shaking, in order to preserve the adhesins. After PBS washing, bacteria were quantified by OD measurement (1.0 OD_600_ = 2×10^9^ CFU/ml). Bacterial adherence on infected cells was confirmed by microscopic observations.

### LPS and adhesin purification

LPS preparations were prepared from the non-pathogenic AAEC185 and wild-type C1845 strains, using the hot phenol-water LPS extraction protocol as previously described by [80]. Bacterial endotoxin was quantified with the *Limulus* amebocyte lysate (LAL) chromogenic assay, according to the manufacturer's instructions (LAL QCL-1000a, Cambrex).

Adhesins were purified as previously described in [27] and quantified on a coomassie-stained SDS polyacrylamide gel.

### Cell culture

The human colon carcinoma cell line T84 was supplied by the ATCC (number CCL-248) 37]. The cells were cultured as a monolayer for 6 to 7 days to confluency in DMEM:F12 medium containing HEPES supplemented with 5% heat-inactivated fetal calf serum (FCS) without antibiotics. The cells were maintained at 37°C in a 5% CO_2_-95% air atmosphere. Experiments were performed on polarized cells (5 days post-confluence). Prior to infection, cells were starved overnight in DMEM:F12 medium containing HEPES without FCS. The Caco-2/TC7 clone, established from the parental human enterocyte-like Caco-2 cell line [81], was grown in DMEM supplemented with 20% heat-inactivated fetal calf serum and 1% nonessential amino acids at 37°C in a 10% CO2-90% air atmosphere. For experiments, fully differentiated cells were used. LS174 human colon adenocarcinoma cells (LS174T) and INT407 human embryonic intestinal cell line isolated from the jejunum and ileum of a human embryo at about 2 months of gestation were obtained from the ATCC, and cultured in the recommended medium.

Human vascular endothelial cells were isolated from umbilical cord veins by collagenase perfusion and maintained in culture as previously described [82].

### Preparation and analysis of RNA

Cells seeded in a 6-well plate were lysed in TRIzol (Invitrogen). RNA was prepared according to the manufacturer's protocol. 20 µg of RNA was used for Northern blot analysis and hybridized with the VEGF probe (640 bp) corresponding to the coding region of mouse VEGF (NCB accession number NM-009505). The 36B4 probe corresponds to the region comprised between bases 683 and 842 of the cDNA coding for the human acidic ribosomal phosphoprotein P0 (NCB accession number M17885).

Real time quantitative amplification of RNA (q-PCR) was performed on cDNA prepared according to the manufacturer's protocol (Omniscript RT kit, Quiagen). Q-PCR on indicated genes was performed as follows. Two µl (5-fold dilution) of extracted cDNA was used as template for PCR amplification in a 7300 Real Time PCR system (Applied Biosystems). The relative expression of indicated transcripts was quantified using the Taq man PCR Master Mix (Eurogentec) and specific primers furnished by Applied Biosystems on an ABI PRISM 7300 Sequence Detection System (Applied Biosystems) using the 2^[−ΔΔC(T)]^ method. According to this method, the C(T) values for the expression of each transcript in each sample was normalized to the C(T) values of the control mRNA (36B4) in the same sample. The values of untreated cell samples were then set to 100% and the percent transcript expression was calculated. Each experiment was done in duplicate. The results are representative of at least three independent experiments. The significance of the results was estimated by performing a Student's t test.

### ELISA of secreted VEGF

C1845-infected cell culture supernatants were collected, centrifuged at 10 000 rpm for 30 minutes and filtered on a 0.22 µM filter to remove all remaining bacteria. Determination of the VEGF concentration of all supernatants was carried out using an ELISA kit Quantikine Human VEGF Immunoassay (R&D Systems) following the manufacturer's guidelines.

### Western blotting

Serum-starved cells seeded in a 12-well plate were stimulated as indicated and immediately lysed in SDS sample buffer. Protein extracts were resolved by SDS-PAGE in a 10% gel and transferred onto a PVDF membrane (Immobilon-P; Millipore). Membranes were incubated overnight in the presence of the indicated antibodies (previously diluted in milk), washed and then incubated in the presence of a second anti-rabbit or anti-mouse horseradish peroxidase-conjugated antibody. Bound antibody was revealed using an ECL system (Millipore). Where indicated, immunoblots were quantified using the GeneGnome chemiluminescent imaging system (Syngene, Frederick, MD).

### Mitogenesis assay

HUVEC were plated into 12-well plates and cultured as above described. The cells were subjected or not to non-infected or wild-type C1845-infected T84 culture medium for 12 h in serum-free media. ^3^H-methyl thymidine (1 µCi) (Amersham Biosciences, Orsay, France) was added to each well and allowed to incorporate for the last 5 h of incubation. The reaction was stopped with ice-cold PBS. The cells were washed with 5% perchloric acid, rinsed with PBS and solubilized in Triton X-100 1%/NaOH 1%. The solubilized cells were assayed for ^3^H using a TRI-CARB model 2300TR liquid scintillation counter (PerlinElmer, Courtaboeuf, France).

### Tube like structure formation

Capillary-like structure formation was analysed on a bed of Matrigel (Beckton Diskinson) (200 µl Matrigel in 35 mm diameter well). The gel was polymerized at 37°C and 5×10^4^ cells in endothelial growth medium were seeded on the matrix. Morphological changes were microscopically monitored.
